# Study on drainage mode and anti-clogging performance of new waterproofing and drainage system in a tunnel

**DOI:** 10.1038/s41598-023-32590-9

**Published:** 2023-04-01

**Authors:** Cong Zhang, Ning Liu, Kun Chen, FangZhou Ren

**Affiliations:** 1 College of Civil Engineering, Guizhou University, Guiyang, 550025 China; 2 Guizhou Provincial Key Laboratory of Rock and Soil Mechanics and Engineering Safety, Guiyang, 550025 China

**Keywords:** Ecology, Environmental social sciences, Natural hazards, Engineering

## Abstract

With an increase in tunnel construction and retention, traditional waterproofing and drainage systems have been unable to meet the needs of tunnels in heavy rainfall areas, and disasters such as tunnel lining cracking, leakage, and even collapse, occur frequently. In order to ensure the safe operation and maintenance of tunnels, this paper analyses the characteristics of the traditional waterproofing and drainage system, and puts forward a new drainage structure through numerical simulation and indoor testing. This structure removes the circular drainage blind pipe and adds a convex shell drainage plate between the waterproof board and the secondary lining. The research shows that the new drainage system greatly decreases the water pressure in the easily blocked area of the drainage structure. With the special surface discharge model, the external water pressure of the lining far away from the blocked area can quickly fall back to the normal level. In addition, the drainage capacity of different waterproof and drainage boards is different. With an increase in support pressure, the drainage capacity decreases; the geotextile decreases the most, followed by the capillary drainage board and then the convex shell drainage board. At the same time, after the muddy water drainage test of the three materials, it is found that the convex shell type drainage plate has the best anti-sludge performance. The research in this paper provides a beneficial attempt for the design of waterproofing and drainage structure of a water-rich karst tunnel, and provides a guarantee for the safe operation and maintenance of the tunnel.

## Introduction

With the construction of more and more karst tunnels, the problem of tunnel leakage has become a big problem for countless tunnel builders. The leakage of tunnel water will cause lining corrosion, track and parts corrosion, tunnel bed mud boiling and other phenomena; these worsen the tunnel operating environment and endanger the durability of the tunnel structure. Examples of this are the Gaotian Tunnel of the Guiyang-Guangzhou Railway, the Hongqiao Tunnel of the Wuhan-Guangzhou Railway, and the small mountain tunnel of the Shanghai-Kunming Railway. Inverted arch deformation and damage caused by high water pressure have occurred on the Kunming-Nanjiang Railway and other places, resulting in serious economic losses. Whether considering the construction or operation process, the treatment of tunnel groundwater is one of the most important issues for structural safety in karst areas. A large number of scholars have conducted relevant research on this issue.

In terms of theoretical calculations, obtained the leakage field distribution in a deeply buried high head tunnel by using the analytical method^1^. Based on Harr’s classical solution of an unlined tunnel, combined with the actual hydrological environment (taking the surrounding rock, grouting ring, and lining as a complete system), the seepage field water pressure equation was derived^2^. Compared various analytical and numerical solutions, and proved the reliability of the analytical solution^3^. Proposed a semi-theoretical analysis method for calculating seepage^4^. Through theoretical analysis, indoor testing and field measurements, the structural form and controllable drainage scheme suitable for a high water level tunnel were proposed^5^. Established a scale model and the results showed that the PWW method can reduce the water pressure and strain of the lining under the drainage condition while, under the free drainage condition, the liner strain using the PWW method can be reduced by about 30%^6,7^. A three-dimensional numerical model was established and it was found that the water pressure in the tunnel vault was low and the inverted arch water pressure was high. For water-rich karst tunnels, the centre of the inverted arch was easy to crack when using semi-enveloping and semi-draining mode^7^. Studied the evolution law of lining water pressure under the action of dynamic water pressure, proposed an optimised drainage scheme to reduce the inverted arch water pressure, and analysed the effect of water prevention and drainage^8^. Taking Gongbei Tunnel as an example, an analytical solution was proposed to calculate the effective stress caused by seepage around a shallow water tunnel in an elastic half-plane. The high water pressure accumulated behind the lining is the main cause of water pressure disasters. In order to determine the water pressure distribution behind the lining^9^, deduced the analytical formula of the surrounding rock grouting lining water pressure and the relationship between the lining water pressure and the permeability coefficient using the axisymmetric analysis method^10,11^. Based on the conformal transformation method, derived the calculation formula of the water pressure on a circular tunnel lining under a steady seepage state^12–14^. Established the analytical solution of water inflow for the stable seepage of a circular tunnel under an isotropic permeability coefficient. In terms of materials^15^, studied the waterproof performance of rubber gaskets from four aspects^16^. Stated that Switzerland, Austria and other countries use polyethylene and polyvinyl chloride as waterproof materials and they are widely used^17^. Developed composite waterproof and drainage materials^18,19^. Introduced a new type of liquid waterproofing material (a water-based permeable crystalline type) and analysed the differences between the combination of this material and shotcrete and formwork concrete, from a microscopic perspective. Capillary and convex shell waterproofing and drainage boards are relatively new drainage materials^20^. Studied the influence of capillary drainage board on the drainage capacity of sandy soil by conducting indoor drainage tests and setting an angle to study its anti-silting performance. The results show that the value range of the angle of capillary drainage belt lying on the subgrade, slope and other structures is recommended to be 10°–15°^21^. Conducted water pressure resistance and durability tests on five different waterstops and applied them in the waterproof system of Gongbei Tunnel. In terms of structural optimisation^22^, Proposed a waterproof and drainage design concept suitable for the East Tianshan Tunnel: the construction technology of “one block, two drainage and three prevention”^23^. Proposed a composite waterproof and drainage system (CWDS). The results of the research showed that, in the case of blind pipe blockage, the water pressure of the traditional drainage system in a tunnel increases rapidly, while the CWDS tunnel can effectively drain and reduce pressure^24^. Proposed the triple optimisation measures of the structure and the research results will play an important guiding role in the design, construction and maintenance of the drainage systems of highway tunnels in China^25^. Developed a drainage seepage model comprising drainage pipes, waterproof membranes and geotextiles. This study is helpful to the optimal design of tunnel waterproofing and drainage systems, such as estimating the initial lining permeability and thickness, the distance between circular drainage pipes, and the hydraulic conductivity of geotextiles^26^. Through numerical simulation and model testing, three optimised waterproofing and drainage schemes were studied, the results showed that, when conventional waterproofing and drainage schemes are adopted for water-rich karst tunnels, the drainage system cannot effectively reduce the water pressure at the inverted arch of the tunnel. When a longitudinal blind drainage pipe was added at the bottom of the inverted arch, the reduction rate reached 84% and when the central drainage ditch was set at the bottom of the inverted arch, it increased to 96%^27^. Proposed a new concept for a drainage and pressure reduction system at the bottom of a railway tunnel, which can efficiently discharge the accumulated water at the bottom of the tunnel and achieve the goal of reducing water pressure^28^. Studied the water pressure distribution behind the lining under different waterproofing and drainage forms, and put forward the optimal layout plan of the waterproof board^29^. Proposed a new concept for actively controlling the waterproofing and drainage design by adjusting the strength and permeability of the surrounding rock, reinforcement ring and the initial support structure. In order to actively and reasonably reduce the tunnel water pressure^30^, proposed a specially designed drainage system with anti-blocking and automatic release of water pressure^31^.

The research above showed that the conventional drainage scheme cannot solve the tunnel water leakage problem in a water-rich karst tunnel. At present, research in the tunnel seepage field mainly focuses on the calculation of the external water pressure of the tunnel lining, the prediction of water inflow, and research on the optimisation measures of the tunnel waterproofing and drainage system, including new technology and new materials. The research into the causes and effects of plugging has not stopped. However, there are few reports on the distribution of water pressure between blind circular pipes, the drainage effect of blind non-circular pipe + convex shell waterproofing and drainage boards, or the effect of local blockages on the external water pressure of tunnel linings. Therefore, this paper proposes a waterproofing and drainage system with convex shell waterproofing board instead of circular blind pipe, and studies the drainage effect of the new waterproofing and drainage structure through indoor testing and numerical simulation.

## Drainage and waterproofing system

### Traditional waterproofing and drainage systems

In tunnel construction, to prevent underground water from encroaching on the tunnel structure, the traditional anti-drainage system is mainly composed of blind circular drainage pipes, blind longitudinal drainage pipes, waterproof plates, sealing strips, central drainage ditches, and side wall drainage ditches, etc. At the end of the tunnel excavation, the initial support is applied and plays a waterproofing role, to some extent. The water seepage from the surface of the initial support is collected by the blind pipe and discharged by the central drain. The waterproof plate and sealing strip prevent the underground water from eroding the lining.

### Deterioration of traditional drainage system

**Figure 1:**
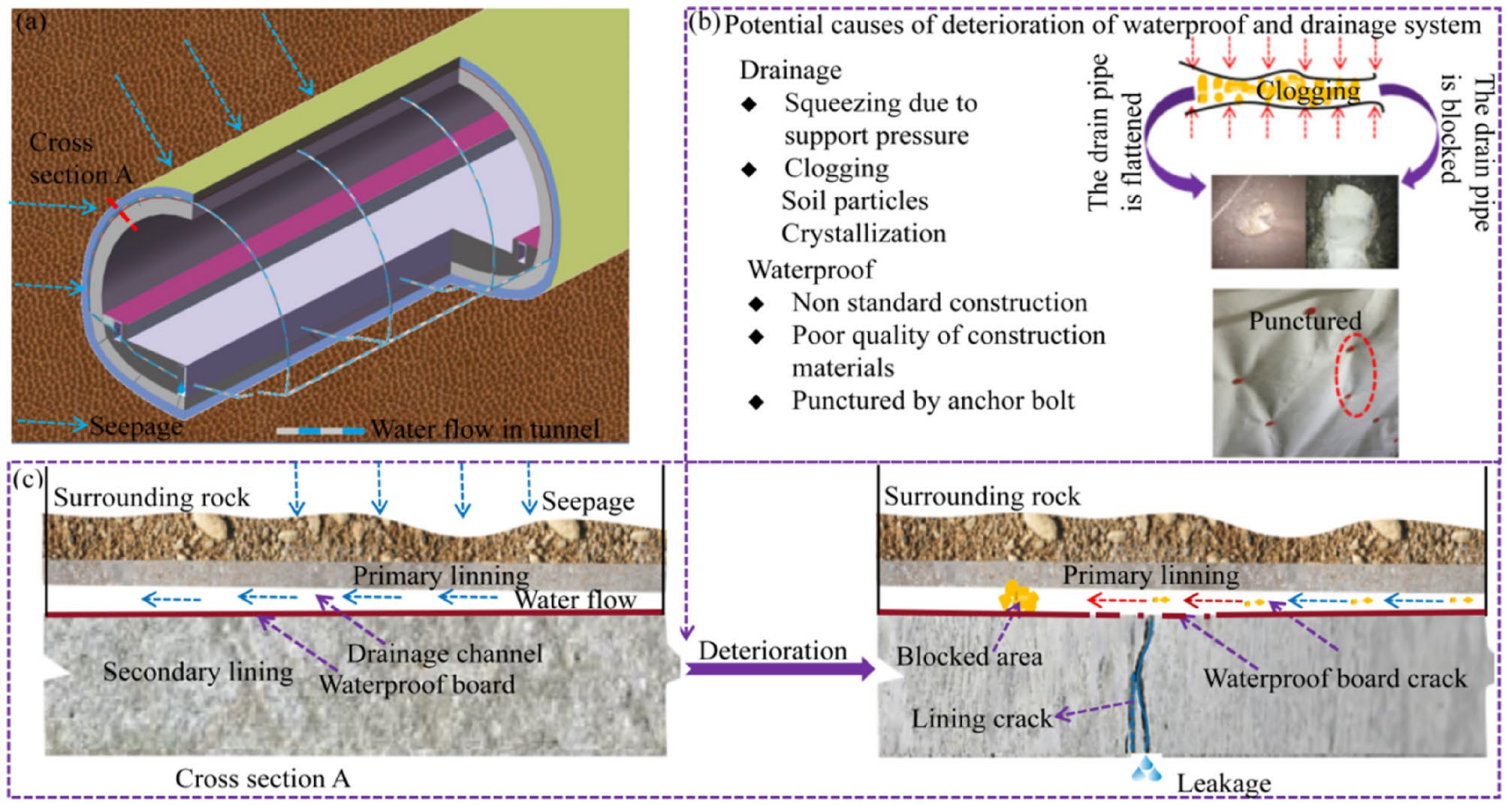
The diagram of tunnel deterioration. (**a**) Water flow in the tunnel; (**b**) Potential causes of deterioration; (**c**) Leakage from the crack of section A.

The traditional anti-drainage system is shown in Fig. 1. The initial support acts as the first layer of waterproofing against groundwater. When the water seeps from the surface of the initial support, the blind drainage pipe will collect and transport the seeped water to the central drain for discharge. However, as shown in Fig. 1b,c, during the tunnel operation, soil particles and chemical crystals will lead to the blockage of the blind drainage pipe. The water pressure in the blocked area will rise sharply, which may lead to the cracking of the lining due to the increased water pressure; the tunnel drainage system will be paralysed and the tunnel operation will be affected. As can be seen from Fig. 1b, the blind drainage pipe is prone to deformation under the action of supporting pressure, which leads to a decrease in drainage capacity. When the wet season commences, the decrease in drainage capacity will lead to a surge of water pressure behind the lining, which will also lead to lining cracking and, finally, tunnel leakage. In addition, there are also higher requirements for the construction personnel; if the construction is improper, the waterproof board will break easily. In summary, with the long-term operation of the tunnel, the drainage system gradually ages, causing tunnel leakage, lining cracking, and other problems.

### Design of new drainage system

**Figure 2:**
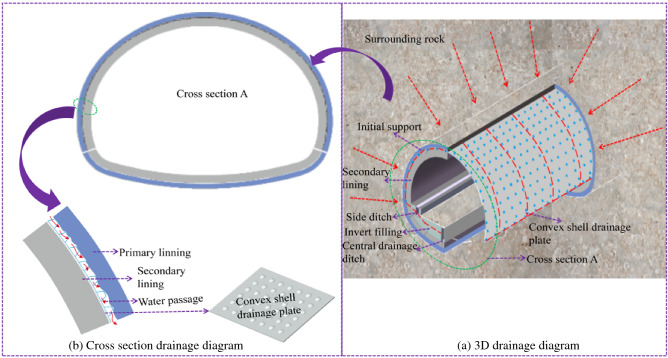
New drainage system.

A new type of drainage system is proposed, to solve problems such as straightforward blockages of the circumferential drain pipe and uneven water pressure distribution after lining and cracking of the waterproof plates. Figure 2a is the three-dimensional structural diagram of the optimised anti-drainage system and Fig. 2b is the cross-sectional drainage diagram of the new drainage system. The convex shell anti-drainage board has the function of complete section drainage. The convex shell is used to form a surface drainage channel between the waterproof board and the initial support, to achieve the effect of annular surface drainage, avoiding the current situation of concentrated circular blind pipe drainage and uneven distribution of water pressure in the upper part of the tunnel, reducing the peak water pressure acting on the upper part of the tunnel.

### Comparison of discharge performance of different drainage systems

**Figure 3:**
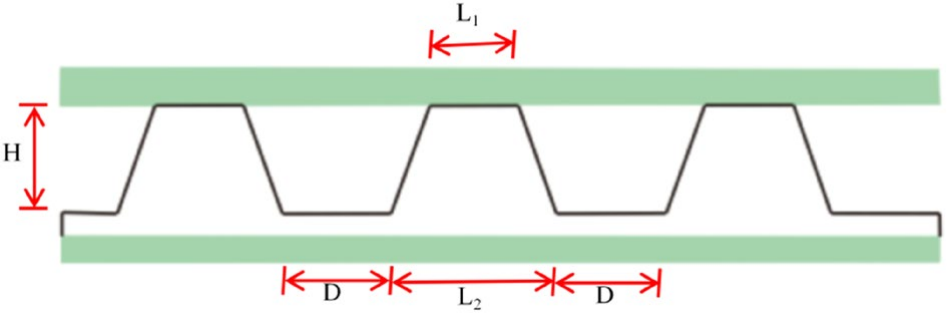
Schematic diagram of convex shell waterproof board section.

Along the axial direction of the tunnel, the water pressure behind the lining is roughly equal, and the theoretical model can calculate the drainage channel. As shown in Fig. 3, the sectional shape of the shell’s anti-drainage board shows the equivalent pipe diameter of the shell anti-drainage channel per square metre of the longitudinal tunnel, which can be calculated according to Eq. (1):

1 $$D_{0} = \left[ {\frac{4}{\pi }\left( {H\left( {1 - \varepsilon } \right)\left( {D + \frac{1}{2}L_{2} - \frac{1}{2}L_{1} } \right)} \right)\left( {\frac{1}{{D + L_{2} }}} \right)} \right]^{0.5}$$

where D_0_ is the equivalent pipe diameter per linear metre of the convex shell waterproofing board’s water passage, m; *H* is the height of the convex hull, m; *D* is the bottom spacing of the convex shell, m; L_1_ is the diameter of the bottom surface of the convex shell, m; L_2_ is the diameter of the convex shell’s top surface, m; ε is the compression deformation rate of the convex shell’s waterproof plate under pressure.

According to fluid mechanics, the equation for calculating the excess water per metre of the waterproof plate is:

2 $${\text{Q}} = 86400\;{\text{CA}}\sqrt {{\text{RJ}}}$$

where ${\text{Q}}$ is the water flow per metre of the shell-like waterproof plate, m^3^/d; A is the area of the pipeline, m^2^; R is the hydraulic radius, m; J is the hydraulic slope; and C is the Xie Cai coefficient. According to Eq. (3), it can be obtained that:

3 $$C = {{R^{{\frac{1}{6}}} } \mathord{\left/ {\vphantom {{R^{{\frac{1}{6}}} } n}} \right. \kern-\nulldelimiterspace} n}$$

In Eq. (3), n is the roughness, depending on the degree of wall smoothness.

When the convex hull height is 1 cm, the compression deformation rate is 0; the convex hull spacing is 0.5 cm, the bottom diameter of the convex hull is 1.6 cm, the top diameter of the convex hull is 0.8 cm and, according to Eq. (1), the equivalent pipe diameter is 7.389 cm and the pipeline area is 42.86 cm^2^.

**Figure 4:**
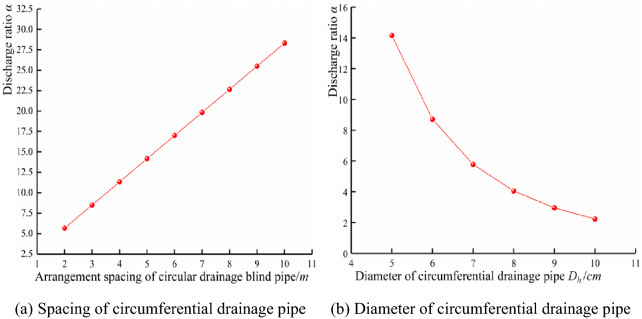
Discharge ratio.

It is known that the diameter of the circumferential drainage pipe of Tiegalishan Tunnel is 5 cm, the spacing of the circumferential blind pipe is 5 m, and the karst and groundwater development area is encrypted to 2 m, assuming that the roughness of the water path is the same. When the hydraulic gradient is the same, the flow ratio can be calculated by Eqs. (1), (2), and (3), as follows:

4 $$\alpha = d\left( {\frac{{D_{0} }}{{D_{h} }}} \right)^{\frac{8}{3}}$$

where α is the flow rate ratio between the annular drainage pipe and the annular drainage pipe with the convex shell waterproof plate in the arrangement spacing; d is the layout spacing of the circumferential drainpipe; and *D*_*h*_ is the diameter of the circumferential drain pipe, m. If the equivalent pipe diameter is 7.389 cm and the annular drainpipe diameter is 5 cm, the relationship between the flow ratio and the spacing layout of the drainpipes is shown in Fig. 4a. When the circumferential spacing is 5 m, the relationship between flow ratio and circumferential drain pipe diameter is as shown in Fig. 4b.

Figure 4a shows that, under the same flow path roughness and hydraulic gradient, the flow ratio maintains a linear increase with the increase of the annular drainage pipe spacing layout. It can be seen from Fig. 4b that, with an increase of annular drainpipe diameter, the flow ratio keeps declining, but the downward trend keeps decreasing. When the equivalent pipe diameter per metre of waterproof shell plate is 7.389 cm, the pipe diameter of the circumferential drainpipe is 5 cm and the spacing of the circumferential drainpipe is 2 m; the excess water ratio is 5.66. When the annular drainpipe spacing layout is 10 m, the flow ratio is 28.33. When the equivalent pipe diameter of the shell waterproof plate per metre is 7.389 cm, the spacing layout of the circumferential drainpipe is 5 m and the diameter of the circumferential drainpipe is 5 cm; the flow ratio is 14.16. When the diameter of the annular drain pipe is 10 cm, the excess water ratio is 2.23. According to the above data, the drainage performance of the convex shell drain plate is better than that of the annular drain pipe.

## Numerical simulation

### Numerical model and boundary conditions

**Figure 5:**
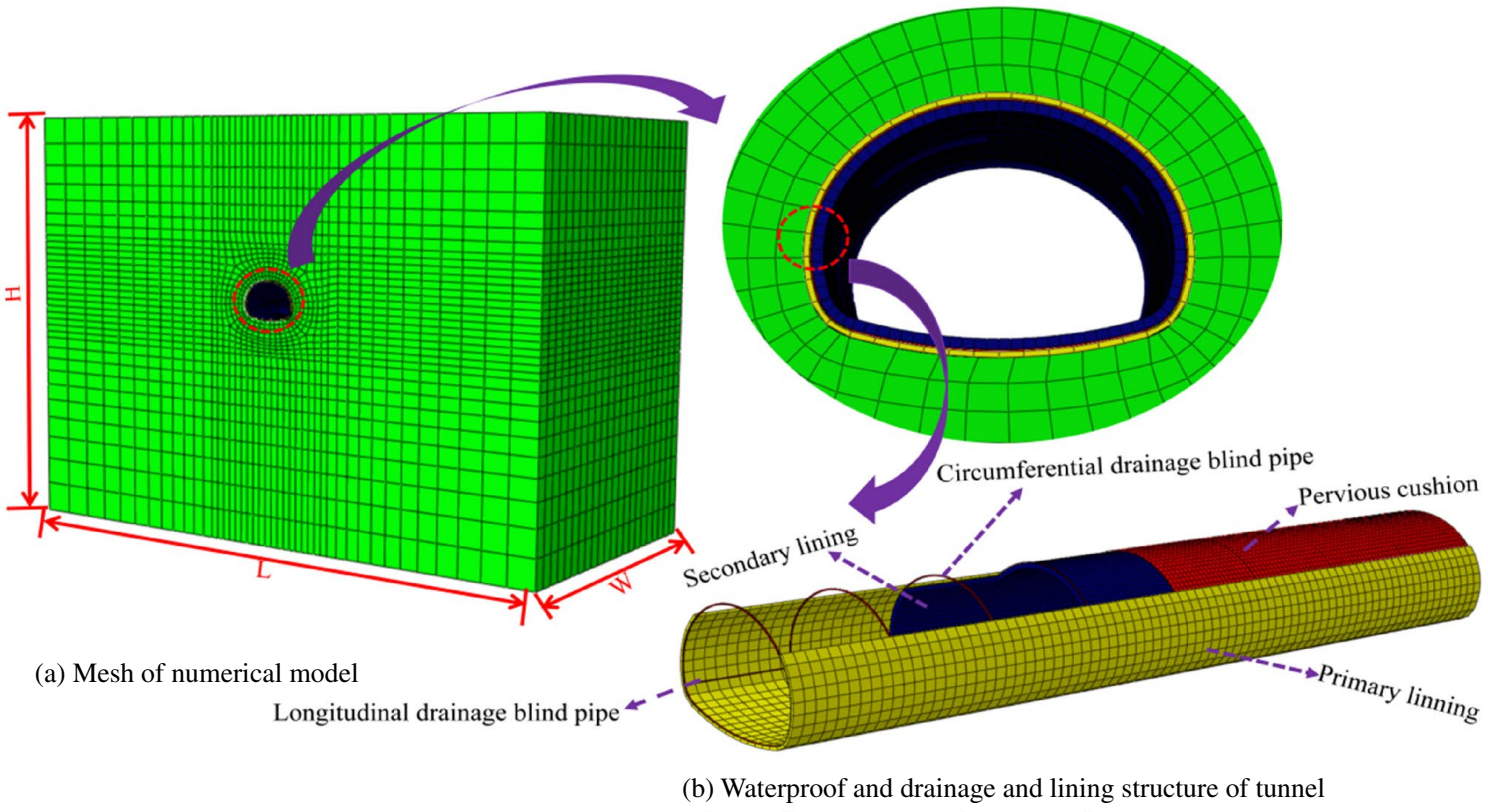
Three dimensional seepage model.

In order to study the distribution law of water pressure after lining the new drainage system and the traditional blind circular drainage pipe drainage system, the changing law of water pressure, after lining at different plugging distances, was applied using ABAQUS software for numerical simulation in a traditional anti-drainage system. The waterproof board and geotextile were set as the anti-drainage layer, which played the role of ‘waterproof’ and ‘drainage’ in the numerical simulation. The permeability of the secondary lining is usually set as an extremely low parameter, to play the role of waterproofing, and the water guide cushion is set to realise the function of ‘drainage’. The water guide cushion is an essential structure for drainage in areas without a blind drainage tube setup and it played the role of replacing the convex shell in this simulation. The model adopted full-section radial grouting to strengthen the water plugging and the thickness of the grouting ring was 5 m. The depth of the tunnel was 45 m, the height of the underground water level was taken from the surface, take 5 times the tunnel diameter from the side wall of tunnel excavation to both sides and about 5 times the tunnel height downward. The longitudinal length was 40 m along the tunnel axis and the model size was 160 m, 40 m wide, and 120 m high. The three-dimensional seepage model of the tunnel is shown in Fig. 5.

Solid element simulation was adopted for the surrounding rock and grouting ring; a More-Coulomb constitutive model was adopted for the mechanical model; the isotropic seepage model was adopted in the fluid model; a solid element was used to simulate the lining, filter layer, and drainage hole; an elastic constitutive model was adopted in the mechanical model; an isotropic seepage model was adopted in the fluid model; physical and mechanical indexes of the surrounding rock were calculated according to the geological prospecting report; and numerical calculation parameters are shown in Table 1.

**Table 1:** Calculation parameters.

Materials	*ρ* (kg/m^3^)	E (GPa**)**	*μ*	*φ* (°)	*c* (MPa)	*n*	*k* (m/s)
Surrounding rock of tunnel	2100	1.5	0.3	35	0.5	0.2	2.0 × 10^−6^
Grouting ring	2400	1.95	0.25	40	0.55	0.15	4.0 × 10^−6^
Primary support	2400	29.4	0.2	–	–	0.07	1.0 × 10^−8^
Pervious cushion	2400	29.4	0.2	–	–	0.1	4.0 × 10^−6^
Secondary lining	2700	31.7	0.2	–	–	0.05	1.0 × 10^−10^
Circumferential drainage blind pipe	2700	31.7	0.2	–	–	0.74	9.4 × 10^−4^

*ρ*, Dry density; E, Elastic modulus; *μ* Poisson’s ratio; *φ*, Internal friction angle; *c*, Cohesion; *n*, Porosity; *k* Permeability coefficient.

### Research on water pressure distribution under normal conditions in a drainage system

**Figure 6:**
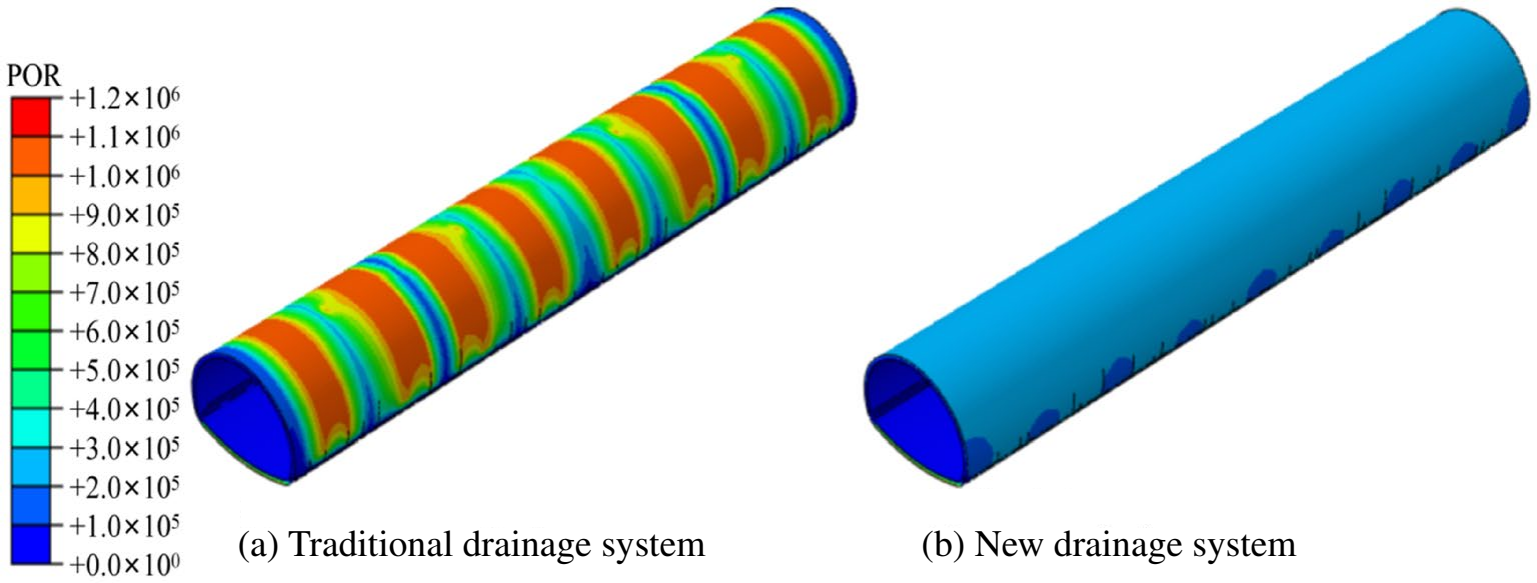
Study on water pressure distribution in different waterproof and drainage systems.

When a tunnel is excavated, the initial support, secondary lining, and corresponding drainage pipeline construction is re-balanced, and the formation of a new stable seepage field will have a new impact on the lining structure of the tunnel. Figure 6 shows the external water pressure cloud diagram of the lining of the traditional and new drainage modes, after the excavation and seepage stability.

**Figure 7:**
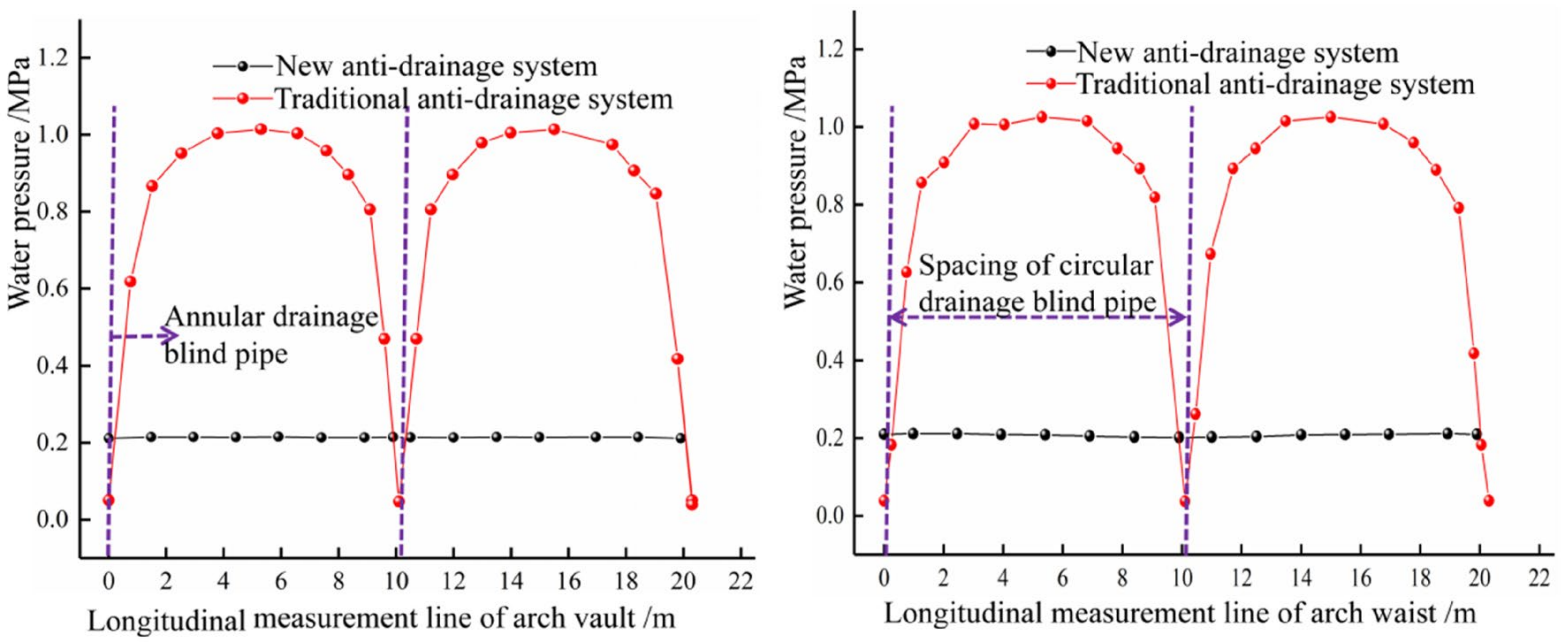
Comparison of water pressure in different drainage modes.

As seen in Fig. 6, the water pressure of the traditional drainage system is slightly near the drain pipe. In contrast, the water pressure in the middle area of the two drains is enormous, presenting a ‘wavy’ water pressure distribution. The main reason is that the blind circumferential drainage pipe has a strong drainage capacity. In contrast, the area without a blind drainage pipe has a weak water transport capacity in its geotextile, resulting in a centralised distribution of water pressure. The new anti-drainage mode eliminates the blind ring pipe and adopts the convex shell waterproof plate. The drainage capacity of the upper part of the tunnel is equal, the water pressure distribution is uniform, and the water pressure is far less than the peak water pressure of the traditional drainage mode, thus realising the transformation from the ‘line discharge’ of the traditional drainage system to the ‘surface discharge’ of the new drainage system. The distribution of the water pressure along the arch and the vault is shown in Fig. 7.

From Fig. 7, the water pressure of the arch and vault in the new drainage mode is roughly maintained at approximately 0.202 MPa and 0.214 MPa. The water pressure is stable and the water pressure gap between the arch waist and the arch crown is small. In the traditional drainage system, the peak water pressure of the arch waist and vault observation line are 1.025 MPa and 1.014 MPa, respectively. The peak water pressure of the new drainage model is reduced by 80.29% and 78.90%, compared to the traditional drainage model.

### Influence of unilateral plugging length of different anti-drainage systems on the external water pressure of the lining

**Figure 8:**
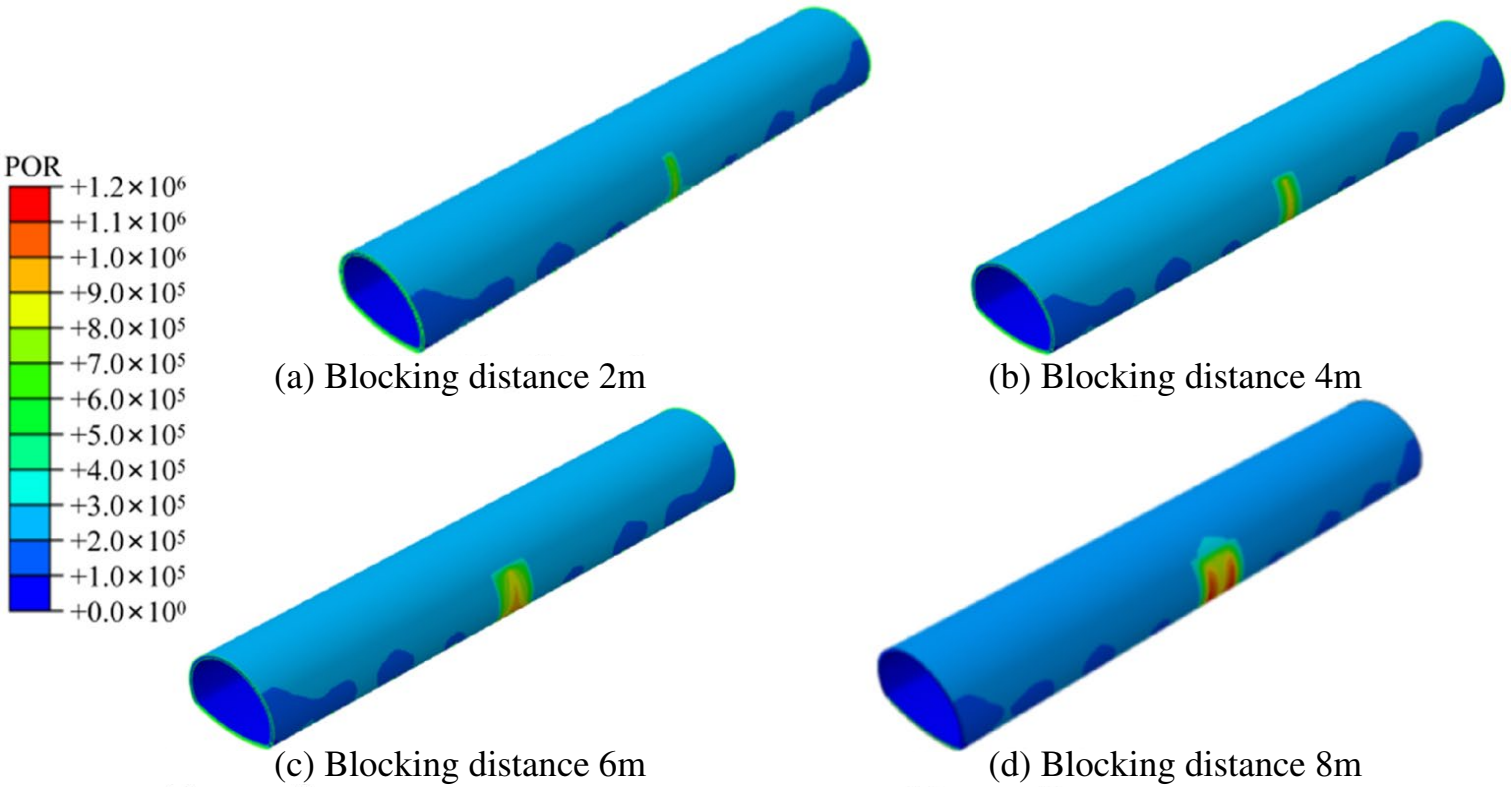
Cloud chart of external water pressure of lining under different blocking lengths on one side.

**Figure 9:**
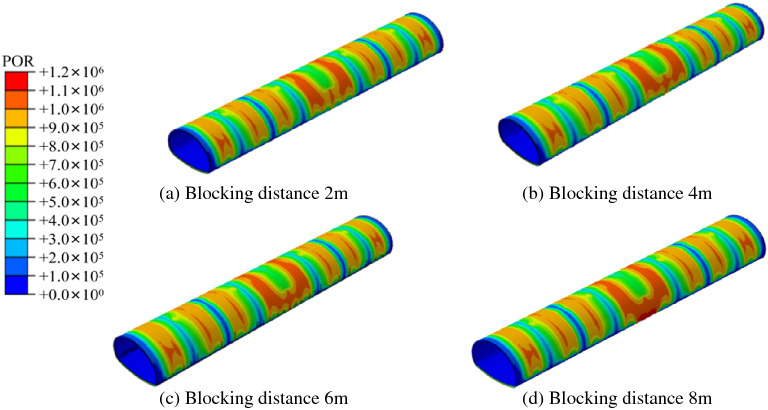
External water pressure cloud diagram of lining with different plugging lengths in the traditional drainage system.

In order to explore the impact of local blockages in different drainage systems on the tunnel lining, four working conditions of 2 m, 4 m, 6 m and 8 m axial blockage of the tunnel side wall were simulated and set. The cloud diagram of the external water pressure distribution of the lining of the new waterproofing and drainage system is shown in Fig. 8. With an increase in blockage length, the water pressure in the blockage area increased continuously. When the blockage length was 2–6 m, the peak water pressure in the blockage area appeared near the midpoint. There was no obvious diffusion phenomenon in the influence range of water pressure along the circumference. When the blocking length reached 8 m, the peak water pressure shifted to both sides, and the influence range of the water pressure began to spread to the unblocked area in the upper part of the tunnel. The cloud diagram of the external water pressure of the lining under different plugging lengths of the traditional drainage system is shown in Fig. 9. With the increase of the plugging distance, the water pressure in the plugging area continued to increase, which is similar to the change rule of the water pressure in the new drainage system. When the drainage system is blocked, the circumferential blind drainage pipe in the plugging area fails.

#### Influence of unilateral blocking length of different waterproof and drainage systems on the external circumferential water pressure of the lining

**Figure 10:**
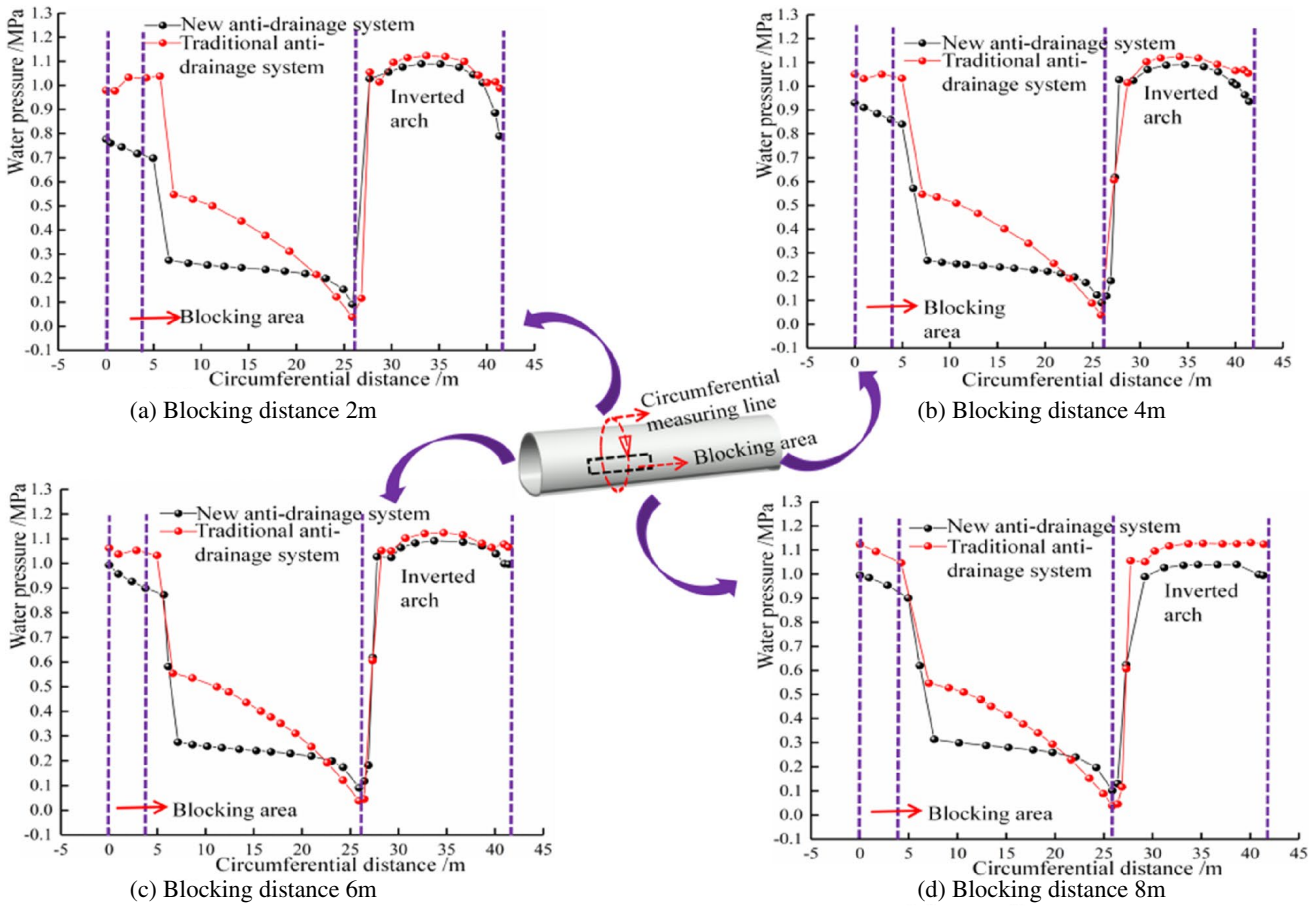
Comparison of circumferential water pressure at different plugging distances.

Figure 10 shows that the water pressure of the traditional drainage system is significantly higher than that of the new drainage system, within the scope of the affected area. In the blocking area, when the blocking length is 2 m, 4 m, 6 m and 8 m, respectively, the maximum water pressure of the new drainage system, along the circumferential direction in the blocking area, is 0.776 MPa, 0.930 MPa, 0.993 MPa and 1.030 MPa. This shows that, the longer the blocked area of the drainage system, the greater the water pressure in the blocked area, but the growth trend slows down. The water pressure outside the lining far from the blocked area will gradually fall back to normal levels but the traditional drainage system will only fall back slowly, while the new drainage system (due to its special surface discharge model) does not rely on the blind circular drainage pipe, so its water pressure will fall back quickly, around the blocked area. The influence range of local blockage on the lining water pressure of the traditional drainage system is also seen to be larger than that of the new drainage system. The influence of local blockage of the new drainage system and the traditional drainage system, on the tunnel’s inverted arch, is relatively limited but the inverted arch water pressure of the new drainage system is slightly smaller than that of the traditional drainage system.

#### Influence of unilateral plugging length of different waterproofing and drainage systems on water pressure of the arch crown

**Figure 11:**
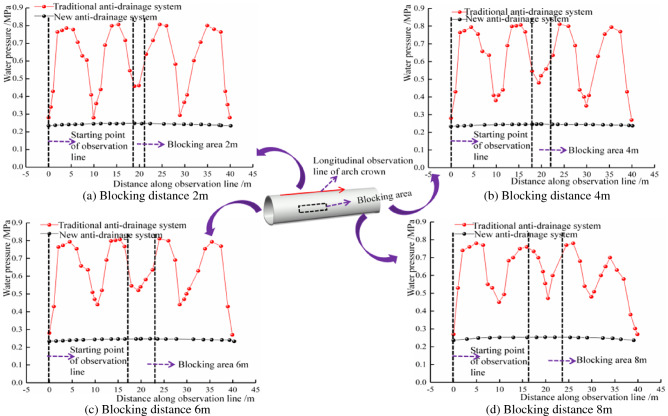
Comparison of water pressure at arch crown under different blocking distances.

The water pressure change curve of the two drainage systems, under different plugging distances, is shown in Fig. 11. For the traditional drainage system, the water pressure of the crown in the plugging area gradually increases with the increase in length of the plugging area, from 0.46 MPa (when the plugging length is 2 m) to 0.54 MPa (when the plugging length is 8 m). The scope of influence is mainly between the blind circumferential pipes on both sides of the plugging area while, for the new drainage system, when a blockage occurs on one side, the water pressure on the arch crown increases slightly. When the blockage length is 2–6 m, the average water pressure is about 0.243 MPa. When the blockage length is 8 m, the average water pressure is 0.248 MPa. The pressure variation of the arch crown is smaller than that of the traditional drainage system.

#### Influence of unilateral blocking length of different waterproofing and drainage systems on water pressure at the arch waist

**Figure 12:**
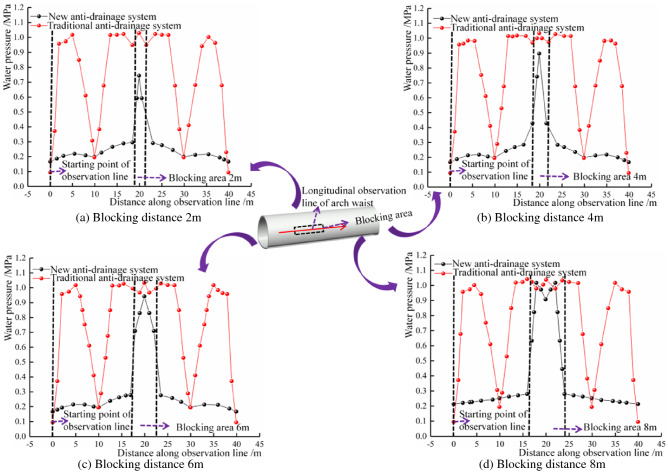
Comparison of water pressure at the waist under different blocking distances.

The water pressure change curve of the two drainage systems under different blockage distances, is shown in Fig. 12. For the traditional drainage system, when the blockage occurs in the layout section of the blind drainage pipe, the drainage function of the blind circular pipe in this area will fail, and its water pressure will rise to the peak water pressure in the middle area of the two blind pipes, under normal drainage conditions. For the new drainage system, it can be seen that the water pressure in the blockage area is significantly increased. When the plugging length is 2–6 m, it shows a single peak (0.744 MPa, 0.897 MPa and 0.942 MPa, respectively). When the plugging length is 8 m, there are two peaks, and the pressure at the midpoint of the plugging area is lower than the peak pressure. The water pressure in the blocked area is smaller than that of the traditional drainage system. In the direction of the increase in the length of the blockage, the influence range of the blocked area is greatly reduced, which is limited to the blocked area. In the periphery of the blocked area, due to the unique ‘surface discharge’ mode of the new drainage system, the water flow can be collected into the longitudinal drainage pipe through the three-dimensional space between the waterproof board and the initial support. The traditional blind circumferential pipe drainage mode mostly relies on the blind circumferential pipe at a certain distance, to collect water. Once the blind drainage pipe is blocked, it means that the spacing of the blind pipe arrangement in the blocked area increases exponentially.

## Experimental study on water performance of tunnel anti-drainage materials

### Overview of drainage materials

The drainage material's performance directly affects the tunnel's normal operation. The existing research shows that the height of the groundwater level in the tunnel increases with a decrease in the drainage performance of the tunnel, which will make the tunnel lining structure bear a more significant load. To truly consider the actual service conditions of drainage boards in tunnels and verify the reliability of numerical simulation, based on relevant test procedures, a water-passing performance test device for tunnel waterproofing and drainage materials was developed, according to the classification of Railway Tunnel Drainage Board (TB/T3354-2014). The materials selected for the testing were geotextile, shell drainage board and capillary drainage board. Geotextile is usually used as a drainage cushion in tunnels. It is used as a buffer layer to protect the waterproof plate; it also has a specific capacity for water guiding, filtering and drainage. To facilitate the comparison of drainage performance, geotextile was added to the tests; the drainage materials tested are shown in Table 2.

**Figure 13:**
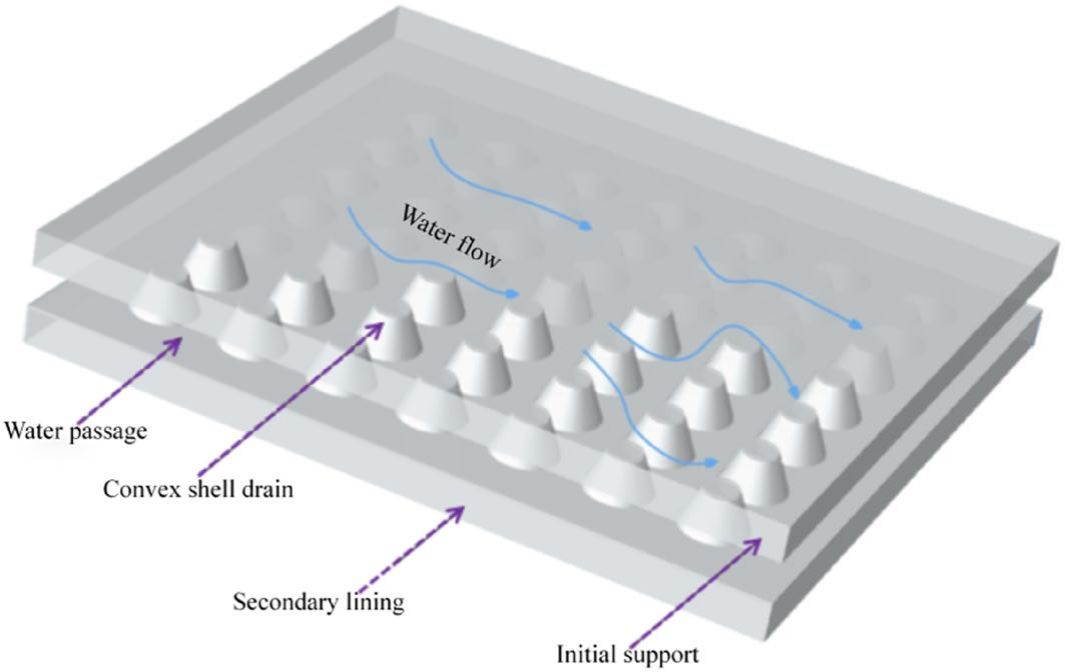
Schematic diagram of water passage of convex shell drain plate.

1. Convex shell drainage plates are mostly made of high-density polythene, with closed convex shells on the surface, usually of a round table or hemispherical shape. When the convex shell’s waterproof plate is attached to the surface of the initial support, the convex shell can provide a certain support height and form a sheet of water passage, as shown in Fig. 13.
2. The capillary drainage board is mainly made of PVC. Under ‘gravity and capillary force’, the water flow is sucked back into the capillary hole’s groove, which fills quickly. Under the action of water surface tension, it forms a closed shape and the installation drop is used to generate ‘siphon force’. The three forces are combined to collect, transport and discharge the groundwater flow.
3. Geotextile materials are mostly synthetic fibres with good water permeability. They are usually used as a drainage cushion between the initial support and the waterproof plate in a tunnel’s anti-drainage system. Geotextile performs the functions of buffering, water guiding, water filtering and drainage.

**Table 2:** Test waterproof material.

Type	Geotextile	Convex shell drain board	Capillary drain board
Physical picture			
Thickness (mm)	4	11	2
Specifications	400 g/m^2^	Shell 10 mm Spacing 5 mm	Aperture 1 mm Thickness 2 mm

### Composition of the test system

**Figure 14:**
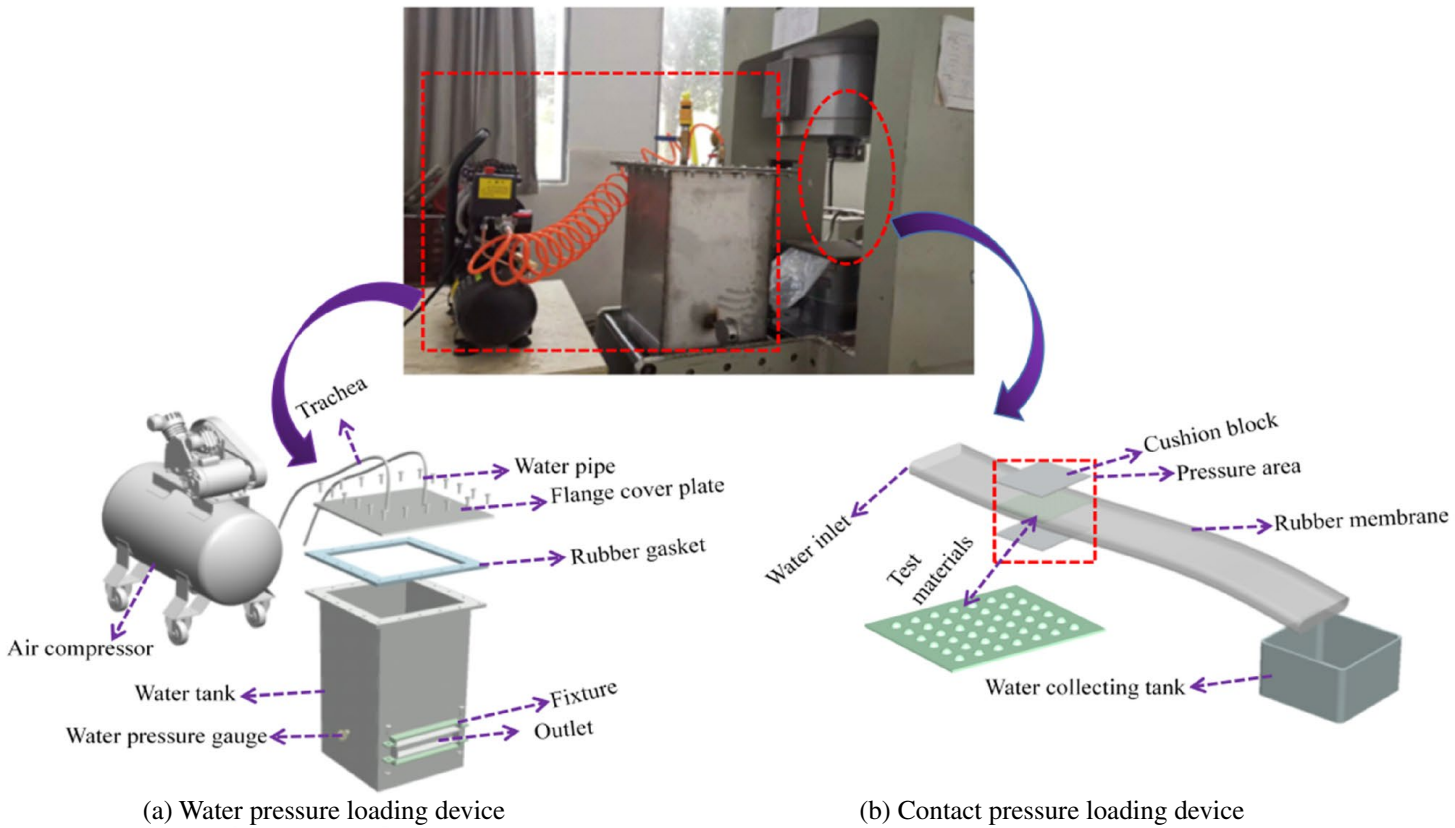
Water passing performance test device.

At present, the drainage performance testing of geosynthetics is mainly conducted according to the instruments and methods given in the ‘Test Procedures for Civil Synthetic Materials of Highway Engineering’ (JTG E50-2006) and the ‘Test Procedures for Geosynthetics’ (SL235-2012). Because of the significant difference between the water head difference and the supporting pressure provided by the instrument and the natural environment of the tunnel, the test results can not reflect the actual situation of the drainage material in the tunnel. Therefore, considering the actual condition of the drainage plate in the tunnel, this experiment is based on the drainage performance test device developed by Chen^32^. This mainly comprises a water storage tank, air compressor, contact pressure loading device, and a water flow collection device, through which the drainage capacity test and anti-blocking capacity test can be carried out, see Fig. 14.

The water storage tank is composed of a steel plate with a thickness of 10 mm and dimensions of 50 × 50 × 100 (length × width × height); the top of the water tank is connected to the water tank with high-strength bolts. A rubber gasket is used between the flange cover plate and the water tank, to ensure the tightness of the water tank. At the top of the water tank, there is a water injection port with an aperture size of 50 mm, through a connection with the water transmission pipe, which provides the acting head in the water tank outlet, at the same height as the water pressure gauge. This keeps a timely record of the laboratory water pressure. An outlet at the bottom of the water tanks allows it to be easily cleaned after the test.

The water pressure loading device is composed of an air compressor, rubber tube and pressure gauge; the water tank can only provide a maximum water pressure of 10 kPa. When the water pressure required for the test exceeds the maximum value that the water tank can provide, the water pressure loading device provides the required water pressure. For the loading of normal pressure onto geotextile drainage materials, according to ‘the Test Procedures for Geosynthetics’ (SL235-2012), the loading plate method is used for the pressurisation; the RMT-301 mechanical test system provided the contact pressure loading device. To ensure that the force of the specimen was uniform and the acting area met the requirements, two rigid pads (of dimensions 25 × 25 cm, i.e. slightly larger than the size of the test piece), were placed on the loading platform to simulate the two-layer support pressure on the drainage plate. The drainage channel and the water tank are connected, the end of the drainage channel is provided with a graduated water collection tank to measure the size of the water outlet, and the above device is assembled into a set of reasonable and feasible, functional drainage performance test devices.

### Test content

To test the drainage performance of geotextile drainage materials under different working conditions, including drainage capacity and anti-silting capacity, so that the test results can guide the tunnel construction according to the hydraulic gradient test in ‘Measurement of Water Flow in Plane of Geotextiles and Related Products’ (GB/T 17633-2019), it can be seen that at least 0.1 and 1.0 hydraulic gradients should be provided. Combined with the depth of the tunnel and the hydrogeological conditions, the maximum water pressure of the test was set as 100 kPa, and the water pressure was set as 20, 40, 60, 80 and 100 kPa. Support pressure can be obtained according to field monitoring and reference to the related highway and railway tunnels; the maximum contact pressure is 500 kPa, so the test is set as 50, 100, 200, 300, 400 and 500 kPa.

#### Drainage capacity test

During the drainage capacity testing, pure water is used to test the drainage performance of the geotechnical materials under different support and water pressures. The test conditions are shown in Table 3.

**Table 3:** Test conditions.

Test materials	Contact pressure (kPa)	Water pressure (kPa)
Convex shell drainage plate	50, 100, 200, 300, 400, 500	0.1, 0.5, 1
Capillary drain board	50, 100, 200, 300, 400, 500	20, 40, 60, 80, 100
Geotextile	50, 100, 200, 300, 400, 500	20, 40, 60, 80, 100

According to the testing procedure’s requirements, the sample's length along the water flow direction should be at least 20 cm, and the width of the sample should be at least 20 cm. During the test, the drainage plate should be cut to 20 × 20 cm and placed in the experimental device; the sample is enclosed in the rubber plate. The sample should be flat without folds, and there should be no leakage around it; the test material and rubber plate must be soaked before the experiment so that the material is saturated. During the testing process, 20 kPa pressure is applied to hold the specimen in place and then test water is injected into the tank to make the water flow from the model box through the drainage channel and drain the bubbles from the specimen. To ensure that the specimen is always in a saturated state during the test, the contact pressure is adjusted to 50 kPa and then, after 15 min, the outlet water flow should be stable. The air compressor is opened to pressurise the water tank to the target pressure, adjusting the water inlet flow, to determine the drainage capacity of the test piece by recording the water displacement over 15 min, and recording when the drainage is stable. The water displaced by the test piece within 15 min is recorded and three groups of tests are conducted under the same working conditions. The average data value is taken as the test data under this working condition.

#### Anti-silting ability test

During the anti-silting capacity test, the test water is replaced by muddy water, prepared by sediment with a particle size of less than 1 mm. A mixer continuously stirs the dirty water to avoid sediment deposition from affecting the test. The sediment mass fraction in the muddy water is 1%. During the test, the contact pressure is 200 kPa and the water pressure is 1 kPa. The drainage volume and sediment content are recorded and used as indicators to evaluate the capacity of the geotechnical drainage materials.

### Drainage capacity analysis of different materials

#### Analysis of water passing performance of drainage materials under different contact pressures

The passing water performance of test materials under different water pressures and supporting pressures is studied by calculating the variation rule of flow rate per unit of time. The calculation formula is as follows:

5 $$q = \frac{{v_{n} }}{{t_{n + 1} - t_{n} }}$$

where *q* is the flow in unit time; $v_{n}$ is the change of water volume in the nth water collecting tank; $t_{n + 1}$ is the end time of the nth acquisition; and $t_{n}$ is the nth acquisition start time.

**Figure 15:**
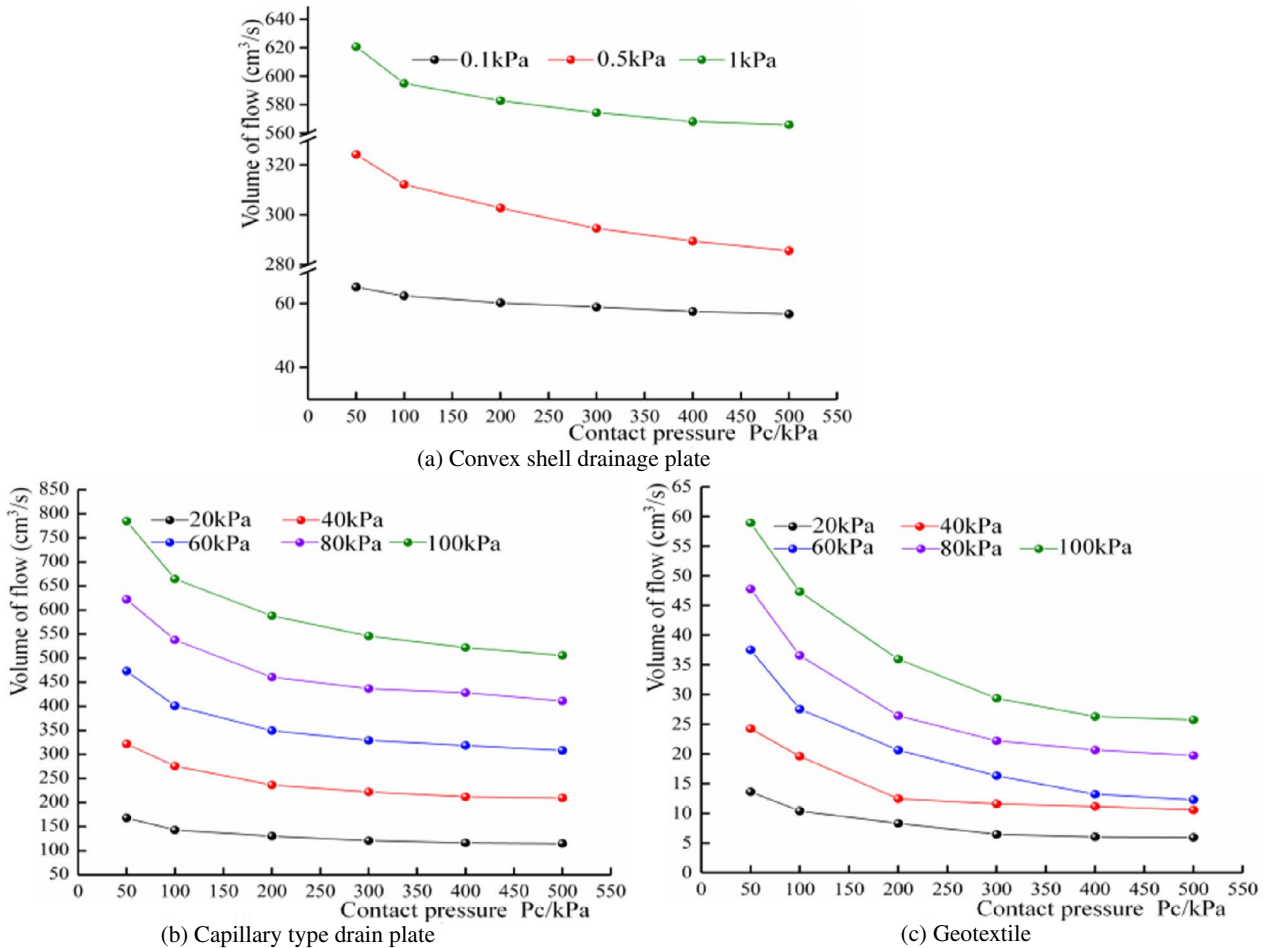
Variation of flow under different contact pressures.

As the maximum inlet water volume of the test is about 1700 cm^3^/s, after many tests on the convex shell drainage plate, it was concluded that, when the water pressure is more significant than 1 kPa, the flow rate has exceeded the maximum inlet water flow. Limited by the inlet flow, to make the test results more convincing, the test water pressure of the convex shell drainage plate was selected as 0.1 kPa, 0.5 kPa and 1.0 kPa; the water-passing performance of the three materials under different contact pressures is shown in Fig. 15. It can be seen that, under the same water pressure conditions, the passing water performance of other materials decreases with an increase in contact pressure. When the water pressure of the shell drainage plate is 1 kPa, and the contact pressure increases from 50 to 500 kPa, the flow rate per unit time decreases from 620.7 to 565.8 cm^3^/s. Drainage performance dropped by 8.8% when the water pressure of the capillary drainage plate is 100 kPa; the discharge rate per unit time decreased from 785.1 to 506 cm^3^/s, with increased contact pressure. The drainage performance decreases by 35.5%, the flow rate per unit time decreased from 58.9 to 25.73 cm^3^/s, in the geotextile, and the drainage capacity decreased by 56.3%. It can be seen that the drainage capacity of the geotextile is most affected by the contact pressures. The effective drainage area of the drain board and geotextile is reduced due to compression and deformation, and the rate of flow reduction is not linear. With the contact pressures increasing from 50 to 200 kPa, the flow rate in unit time decreases rapidly. When the contact pressures increase from 200 to 500 kPa, the rate of flow reduction in unit time is limited.

#### Analysis of material water passing performance under different water pressures

**Figure 16:**
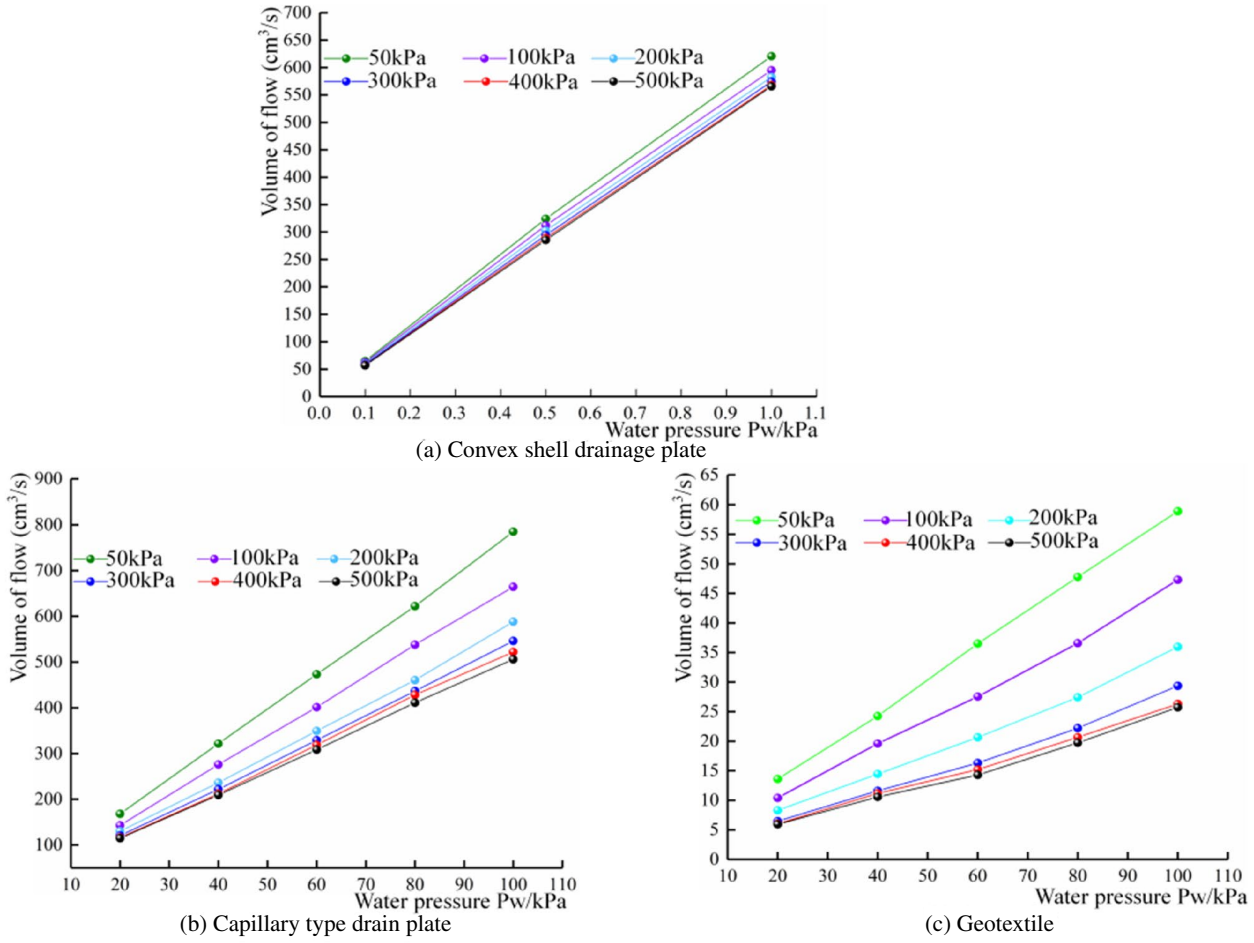
Variation of flow under different water pressures.

The water discharge of the capillary drain plate, convex shell drain plate and geotextile under different water pressure conditions, is shown in Fig. 16. It can be seen that, under the same contact pressure, the water discharge of each drainage material increases with the increase of water pressure and generally maintains a linear relationship. Under the same contact pressure, the deformation of each material remains unchanged, its water passage space remains intact, and the water pressure mainly determines the water discharge.

### Analysis of anti-silting capacity of different drainage materials

**Figure 17:**
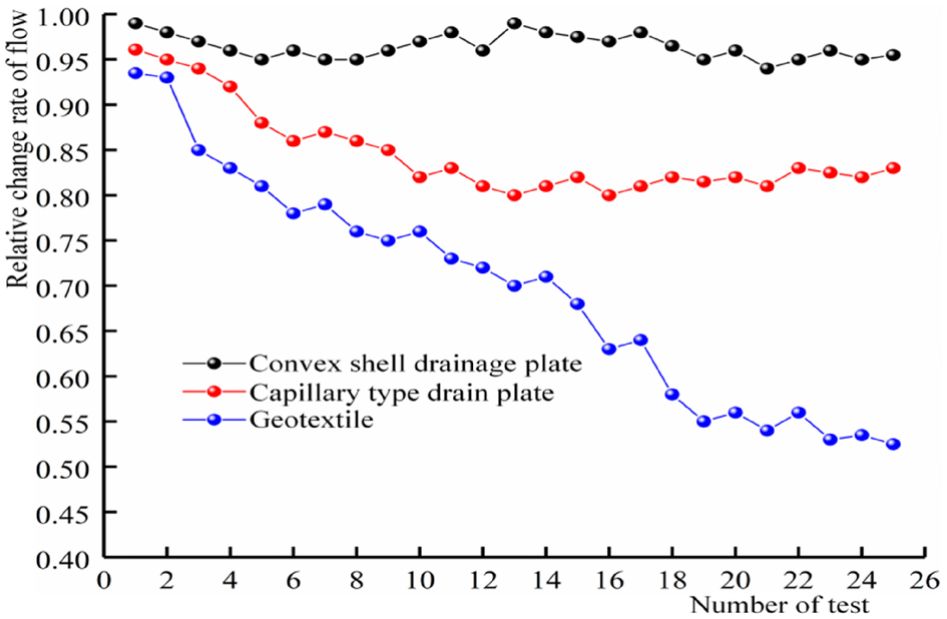
Flow change curve.

To ensure the long-term stable drainage performance of the drainage system, the drainage materials should have good anti-silting capacity. During the operation of the tunnel, due to the seepage of surface water and groundwater, some hydration products in the concrete structure will dissolve in the water and react with the dissolved carbon dioxide to form calcium carbonate precipitation, which can block the drainage pipes. This can lead to an increase in water pressure in the lining, which may then crack. With time, the cracks continue to increase, leading to sediment entering the drainage system through the cracks via water seepage, resulting in silting-up of the drainage system. To study the anti-silting capacity of geotextile, capillary drain boards and convex shell drain boards, a muddy water test is adopted and the drainage change rate (in unit time) is shown in Fig. 17.

**Figure 18:**
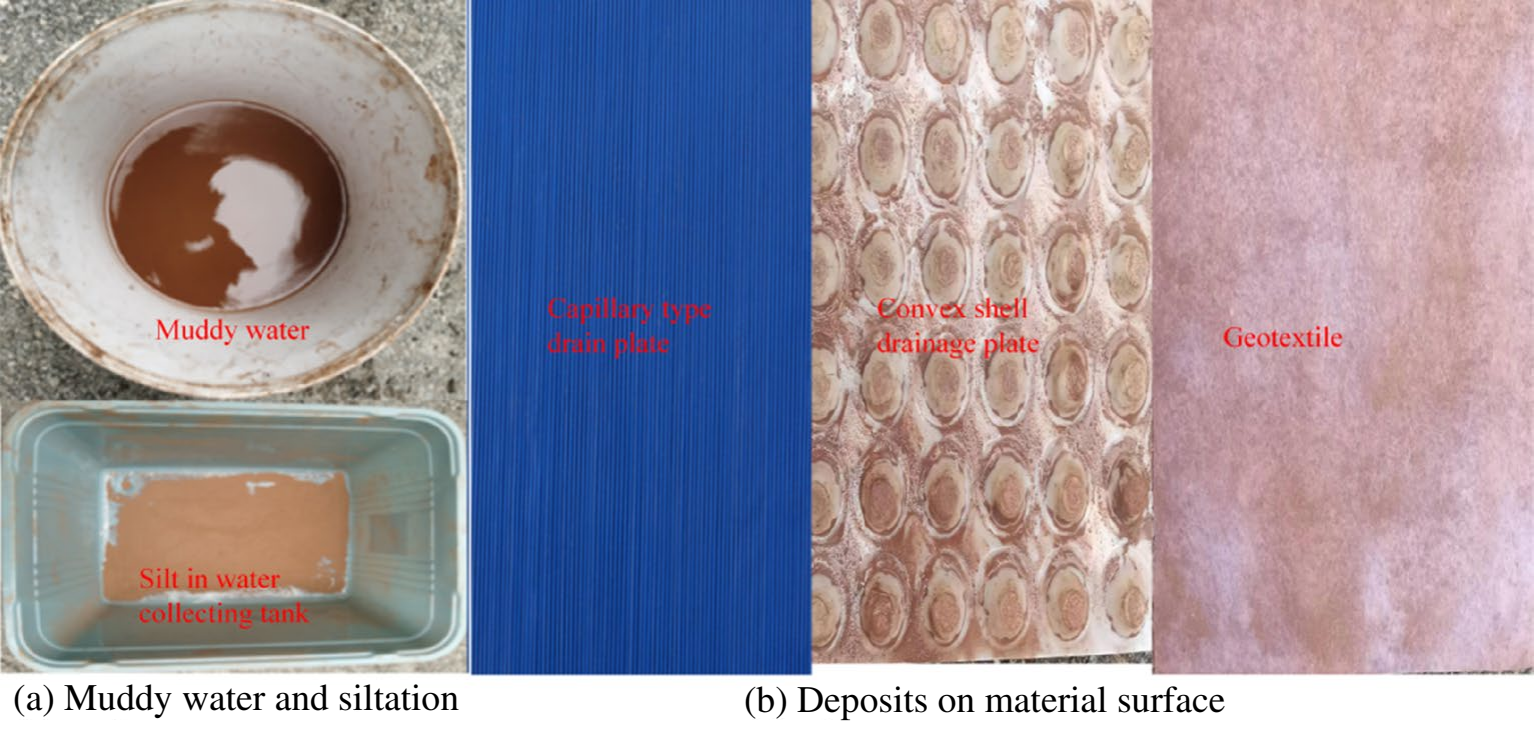
Material anti silting display.

As can be seen from Fig. 18, the drainage capacity of the convex shell drainage plate has yet to be significantly attenuated. After several drainage tests, the relative rate of change of its displacement is still greater than 95% and that of the capillary drainage plate is still greater than 80%. On the contrary, the drainage capacity of the geotextile decreases continuously, and the relative rate of change of the displacement is only about 50% after 25 tests. During the test, the drainage of the capillary drain plate is clear but the convex shell drain plate drainage is turbid. After the test, the sediment in the water collecting tank is shown as in Fig. 18a. After the test, the geotextile and drain plate are removed from the test device, as shown in Fig. 18b.

At the end of the test, the water collection tank and the sample were dried and weighed, and the sediment mass in the water tank and on the material's surface was obtained, as shown in Table 4.

**Table 4:** Sediment quality.

Test materials	Convex shell drainage plate	Capillary drain board	Geotextile
Silt in water collecting tank (g)	362.7	11.3	35.2
Deposits on material surface (g)	18	3.2	29.7

Table 4 shows that, after 25 muddy water tests, the sediment content in the tank reached 362.7 g and the sediment accumulation on the material surface reached 18.3 g. This is because, under the same water head gradient, the discharge of the shell drainage board is large and the sediment particles will flow with the current. The flow of water makes it easier for fine and coarse particles to enter the drainage system, causing the deposition of particles. The convex shell drainage board and the geotextile is covered with a layer of sediment particles on the surface. During the long-term drainage process, the fine particles of the geotextile very easily enter the geotextile and are adsorbed on the pores, which leads to a sharp decline in the permeability of the geotextile and a continuous decrease in drainage efficiency over time. For the capillary drain plate, the width of the capillary groove is only 0.3 mm, which makes it impossible for coarse particles to enter the drain plate and only water flow and a small number of fine particles enter the drainage channel inside the drain plate. However, this will also cause coarse particles to be gradually deposited at the end of the drain plate, which will reduce the discharge area of the capillary drain plate and this shows that the drainage capacity decreases after many tests.

## Conclusion

Based on an in-depth investigation of domestic and foreign literature, and a thorough combination of theoretical analysis, numerical simulation, and indoor experiments, the distribution characteristics of external water pressure in the lining of water-rich karst tunnels and a new waterproofing and drainage system, are studied. The following main conclusions are drawn:

1. The water pressure of the traditional drainage system is small near the drainage pipe. The water pressure in the middle area of the two drainage pipes is large, showing a ‘wave’ water pressure distribution. The water pressure of the new drainage mode is evenly distributed in the upper part of the tunnel and the water pressure is far less than that of the traditional drainage system. The ‘line discharge’ of the traditional drainage system is transformed into the ‘surface discharge’ of the new drainage system. The upper water pressure of the new drainage system is reduced by 80.29% and 78.90%, respectively, compared with the peak water pressure of the traditional arch waist and arch crown.
2. According to the numerical calculation results, the water pressure in the blockage area of the traditional drainage system is significantly higher than that of the new drainage system. When the blockage length is 2 m, 4 m, 6 m and 8 m, respectively (due to its special surface drainage model), the external water pressure of the lining away from the blockage area of the new drainage system will gradually fall back to normal levels. The traditional drainage system will slowly fall back and the maximum circumferential water pressure of the new drainage system will be 0.776 MPa 0.930 MPa, 0.993 MPa and 1.030 MPa. The maximum circumferential water pressure of the traditional drainage system is 1.03 MPa, 1.06 MPa, 1.07 MPa and 1.10 MPa, respectively. The average water pressure at the arch crown of the new drainage system is 0.24 MPa, the average water pressure at the arch waist is 0.873 MPa, the average water pressure at the arch crown of the traditional drainage system is 0.53 MPa, and the average water pressure at the arch waist is 1.06 MPa. Therefore, the water pressure in the blocked area of the traditional drainage system is significantly higher than that of the new drainage system.
3. When the water pressure of the convex shell drain plate is 1 kPa and the contact pressure is increased from 50 to 500 kPa, the flow rate per unit time decreases from 620.7 to 565.8 cm^3^/s, and the drainage performance decreases by 8.8%. When the water pressure of the capillary drain plate is 100 kPa, the flow rate per unit time decreases from 785.1 to 506 cm^3^/s, and the drainage performance decreases by 35.5%; The flow rate per unit time of geotextile decreased from 58.9 to 25.73 cm^3^/s, and the drainage capacity decreased by 56.3%. It can be seen that the drainage capacity of the geotextile is the most affected by the contact pressure, followed by the capillary drainage plate; the effective drainage area of the capillary drainage plate and geotextile decreased sharply due to compression deformation.
4. After 25 muddy water drainage tests, the drainage capacity of the convex shell drain board decreased from 99 to 94%: a decrease of 5%. The drainage capacity of the capillary drainage board decreased from 97 to 81%: a decrease of 16%. The drainage capacity of the geotextile decreased the most, from 94 to 50%: a decrease of 44%. Due to the large porosity of the convex shell drainage plate, the coarse and fine particles will enter the drainage system more easily under the action of water flow, resulting in particle siltation. During the long-term drainage process of the geotextile, the fine particles very easily enter the geotextile and are adsorbed on the pores, which leads to the continuous decline of the drainage in the geotextile over time. For the capillary drainage plate, the width of the capillary groove is only 0.3 mm and so the coarse particles cannot enter the inside of the drainage board; only water flow and a small amount of fine particles enter the drainage channel inside the drainage board. However, this will also lead to the coarse particles gradually silting-up the end of the drainage board, which will reduce the water discharge area at the end of the capillary drainage board, as is shown in the reduction of the drainage capacity after many tests.
5. Under the same contact pressure, the water capacity of the drainage materials increases with the increase in water pressure, and generally maintains a linear relationship. Under the same water pressure, the water capacity decreases with the increase in contact pressure, and the degree of reduction decreases gradually.

## Data Availability

All data generated or analysed during this study are included in this article.
